# Interactive effects of the *APOE* and *BDNF* polymorphisms on functional brain connectivity: the Tasmanian Healthy Brain Project

**DOI:** 10.1038/s41598-021-93610-0

**Published:** 2021-07-15

**Authors:** Manuela Pietzuch, Aidan Bindoff, Sharna Jamadar, James C. Vickers

**Affiliations:** 1 Wicking Dementia Research and Education Centre, University of Tasmania, TAS, 17 Liverpool Street, Private Bag 143, Hobart, 7000 Australia; 2 Turner Institute for Brain and Mental Health, Monash University, Melbourne, Australia

**Keywords:** Genetic interaction, Genetic markers, Genotype, Biomarkers, Diseases, Diagnosis, Disease prevention, Brain imaging, Magnetic resonance imaging

## Abstract

Resting-state functional magnetic resonance imaging measures pathological alterations in neurodegenerative diseases, including Alzheimer’s disease. Disruption in functional connectivity may be a potential biomarker of ageing and early brain changes associated with AD-related genes, such as APOE and BDNF. The objective of this study was to identify group differences in resting-state networks between individuals with *BDNF* Val66Met and *APOE* polymorphisms in cognitively healthy older persons. Dual regression following Independent Components Analysis were performed to examine differences associated with these polymorphisms. *APOE* ε3 homozygotes showed stronger functional connectivity than *APOE* ε4 carriers. Males showed stronger functional connectivity between the Default Mode Network (DMN) and grey matter premotor cortex, while females showed stronger functional connectivity between the executive network and lateral occipital cortex and parahippocampal gyrus. Additionally, we found that with increasing cognitive reserve, functional connectivity increased within the Dorsal Attention Network (DAN), but decreased within the DMN. Interaction effects indicated stronger functional connectivity in Met/ε3 carriers than in Met/ε4 and Val/ε4 within both the DMN and DAN. *APOE*/*BDNF* interactions may therefore influence the integrity of functional brain connections in older adults, and may underlie a vulnerable phenotype for subsequent Alzheimer’s-type dementia.

## Introduction

Alzheimer’s disease (AD) is the most common form of ageing-related dementia, accounting for 60 to 80% of all cases^1^. The primary functional manifestations of AD include memory loss, impairment in executive functioning, difficulties with language, and changes in personality and behaviour, with brain pathology characterized by neurofibrillary tangles and amyloid-beta deposition^1,2^. Neuroimaging techniques investigating activity within and between resting-state networks, such as the default mode network (DMN)^3^, the dorsal-attention network (DAN)^4^, and salience network (SN)^5^, may help provide an understanding of the elementary brain changes that are associated with ageing and subsequent risk of AD. In this regard, significant ageing-related changes in functional connectivity have been observed within the DMN^6,7^. Many studies have focused on the role of the DMN in AD, with some studies showing increased functional connectivity^8^, while others showed decreased functional connectivity^6,7,9^. A further interesting network is the DAN, which is activated during goal-directed behaviour^4^. The DAN showed significant decline in functional connectivity in the amnestic form of mild cognitive impairment (MCI) and in AD^10^ compared to neurologically healthy individuals. This disturbance in connectivity increases with disease progression. Similar results were found within the SN, in which reduced grey matter volume and disrupted functional connectivity were found in patients with AD. Moreover, it was found that healthy older individuals had intra-network functional connectivity impairments between crucial nodes, such as the DMN, indicating that the SN is affected by normal ageing before manifestation of AD^5^. The SN is involved in incoming information processing and filtering information^11^, and is active in higher-order processing such as selecting specific stimuli^12^.

Both life-course and genetic factors may influence susceptibility to ageing and AD-related changes in functional connectivity. Cognitive reserve, for example, is a theoretical construct related to the preservation of brain functionality relative to accumulating degenerative brain changes and pathological lesions^13^. Cognitive reserve is an example of a potentially modifiable risk factor for cognitive decline through education and other cognitively stimulating activity^14^ and may influence functional brain organization. Other possible factors that may influence functional connectivity are gender differences. When comparing males and females’ economic position, men have a superior status in the socio-economic world with higher income and better education, however, women have a higher life expectancy (Carmel, 2019). Nowadays, more women take up tertiary education. These different lifestyles may influence the connectivity in the brain, therefore, exploratory we wanted to investigate whether there are differences in resting-state functional connectivity. Further well-known factors that can influence functional connectivity are variations in genes associated with risk of ageing and AD-related cognitive decline.

For example, the apolipoprotein E gene (*APOE*) ε4 allelic variant is known to be a major risk factor for late-onset AD as compared to the ε3 and ε2 alleles^15^. With respect to functional connectivity, it is unclear if healthy older adult ε4 carriers show changes in DMN functional connectivity compared to ε3 homozygotes. Studies variably show decreased DMN connectivity^16,17^ or increased connectivity^18^; whereas others found no difference between ε4 carriers and ε3 homozygotes^19^.

A common variation is the gene encoding brain-derived neurotrophic factor (*BDNF* Val66Met), a protein important for neurogenesis and synaptic plasticity, has also been investigated for its potential role in brain ageing and AD-related decline. Decreased memory function and reduced hippocampal volume have been described in Met allele carriers compared to Val homozygotes^20,21^. Resting-state functional connectivity has been reported to be relatively decreased in the hippocampus in middle-aged^22^ and older individuals ^23^ who are Met carriers. Such studies have also demonstrated increased functional connectivity in the dorsal lateral prefrontal cortex and anterior insula of Met carriers in a younger healthy population compared to Val homozygotes^24^. Interactions between the APOE and BDNF gene variants may also be possible, as *APOE* ε4 and *BDNF* Met carriers may show relatively increased ageing-related episodic memory impairment^25^.

The current study examined the potential individual and interactive roles of the BDNF and APOE gene variants relative to functional connectivity of resting-state networks in subjects purposively sampled from the cohort study, the Tasmanian Healthy Brain Project (THBP). Subject-specific maps were used to examine age-related differences in functional connectivity addressing the DMN, DAN and SN. We hypothesized that the connectivity of the selected resting-state networks will differ between the *APOE* (ε3 & ε4) and between the *BDNF* Val66Met polymorphisms, and we also predicted interaction effects between these polymorphisms based on the findings of Ward et al. (2014) in this cohort. In particular, we expected to see lower functional connectivity in Met carriers and ε4 carriers compared to Val homozygotes and ε3 homozygotes. All analyses were controlled for age, cognitive reserve, GM maps. Finally, we investigated whether cognitive reserve influenced functional connectivity in the three aforementioned networks, controlled individually for each analysis for BDNF and APOE.

## Results

The current study included only ε3 homozygotes and ε3ε4 carriers. One subject was excluded due to recurring errors within the single-subject independent component analysis, and one subject was excluded due to structural abnormalities in the brain. Participant demographics and characteristics of the 76 THBP subjects comprising this study population are provided in Table 1. *APOE* and *BDNF* polymorphisms were balanced between genders. In particular, a chi-square test was performed to explore the relationship between *APOE* and *BDNF* genotypes and gender. The results showed that our cohort proportions between *APOE* ε3 homozygotes and ε4 carriers, and between *BDNF* Val homozygotes and Met carriers were balanced with no significant differences in proportion. Family-wise error corrections for multiple comparisons using threshold-free cluster enhancement^26^ was used to control Type 1 error rate when investigating differences between *BDNF* Met vs *BDNF* Val, *APOE* ε4 vs *APOE* non-ε4; and the interactions between Met/ε3, Met/ε4, Val/ ε3, Val/ε4.

**Table 1:** Seventy-six participants characteristics expressed as mean (M) ± standard deviation (SD) unless otherwise noted.

	Met	Val	*p*-value	ε4	ε3	*p*-value
Gender (F : M)	25:10	27:14	0.61	23:11	29:13	0.90
Intervention (*N*) (Ex : Con)	28:7	31:10	0.65	26:8	33:9	0.83
Age (years)	63 ± 5.69	63.6 ± 6.82	0.71	63.3 ± 5.15	63.3 ± 7.14	0.64
Education (years)	11.1 ± 0.94	11.4 ± 1.11	0.35	11.2 ± 1.13	11.3 ± 0.97	0.84
Cognitive reserve (*z*)	0.20 ± 0.82	0.16 ± 0.88	0.84	0.06 ± 0.95	0.28 ± 0.76	0.27
Language (*z*)	0.01 ± 0.93	0.16 ± 0.92	0.47	0.11 ± 1.01	0.08 ± 0.87	0.92

Data represented are mean values (*SD*) for continuous variables and proportions for categorical variables. Met and Val relate to participants with specific *BDNF* Val66Met polymorphisms. ε4 + and ε4- refer to participants of the *APOE* polymorphism.

F = female; M = male; Ex = Experimental; Con = Controls; *z* = z score.

### Resting-state network identification

The group independent component analysis (ICA) output allowed us to identify networks from 25 independent components. Within all independent components, we detected 12 networks that have been defined in healthy individuals^27–30^. Identified networks comprised the precuneus together with the DMN, SN, DAN including the left & right dorsal-/ lateral ventral stream, as well as the visual networks (medial, lateral), sensory-motor network, executive control network, auditory network, and cerebellum network (Supplementary Fig. 1).

### Multivariate voxel-based analyses within resting-state networks of the polymorphisms

**Figure 1:**
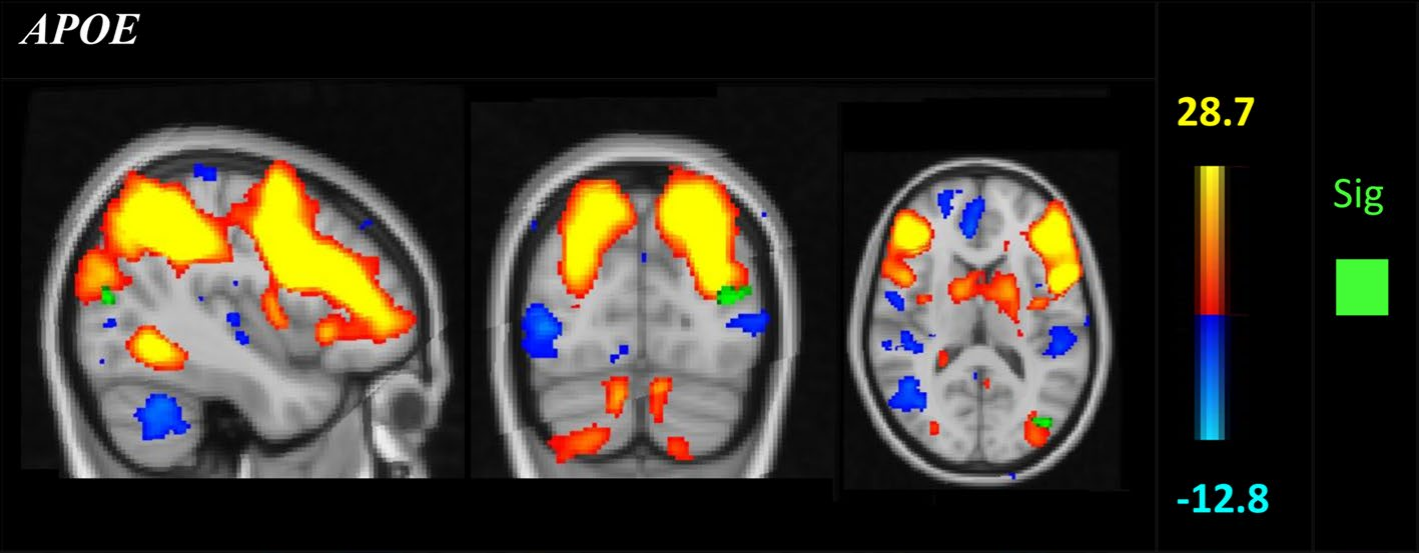
Red-yellow represent the dorsal attention network (DAN) after performing a group-ICA. Green voxels show increased functional connectivity in ε3 homozygotes compared to ε4 carriers in the occipital cortex (*p* < 0.05 family-wise error [FWE]-corrected). Montreal Neurological Institute (MNI) coordinates x = − 42, y = − 80, z = 16. Results were controlled for age, cognitive reserve, and GM maps and the significance was still there. Data was analysed with FMRIB Software Library^31^ and displayed in FSLeyes (https://git.fmrib.ox.ac.uk/fsl/fsleyes/fsleyes/).

After performing a dual regression for each *APOE* and *BDNF* polymorphisms, there was a significant difference in functional connectivity between DAN and occipital cortex, superior division, where *APOE* ε3 homozygotes showed stronger functional connectivity compared to ε4 carriers, *p* = 0.02, (*p* = 0.04 after controlling for age, *p* = 0.03 after controlling for gender, *p* = 0.04 after controlling for cognitive reserve, Fig. 1, Table 2). The dual regression for the *BDNF* Val66Met polymorphisms revealed no significant differences between Met carriers and Val homozygotes.

**Table 2:** Apolipoprotein E (*APOE*) results of analyses after running dual regressions using GLMs.

Cluster Index	Voxels	X (vox)	Y (vox)	Z (vox)	*1-p*	*p*-value	Location
***APOE* (controlled for GM maps only)**							
DAN (ε3 > ε4)							
1	63	66	23	44	0.98	.02	Lateral occipital cortex (superior and inferior division)
***APOE* (controlled for age and GM maps)**							
1	40	65	24	44	0.962	.038	Lateral occipital cortex (superior and inferior division)
***APOE* (controlled for gender and GM maps)**							
1	64	65	24	44	0.974	.026	Lateral occipital cortex (superior and inferior division)
***APOE* (controlled for cognitive reserve and GM maps)**							
1	36	66	23	44	0.963	.037	Lateral occipital cortex (superior and inferior division)
***APOE* (controlled for gender, age, cognitive reserve, & GM maps)**							
1	9	65	24	44	0.952	.048	Lateral occipital cortex (superior and inferior division)

GLMs = General Linear Models; DAN = Dorsal Attention Network; APOE = Apolipoprotein E; ε3 = ε3ε3 homozygotes; ε4 = ε3ε4 carriers; GM = Grey matter.

### Gender differences

**Figure 2:**
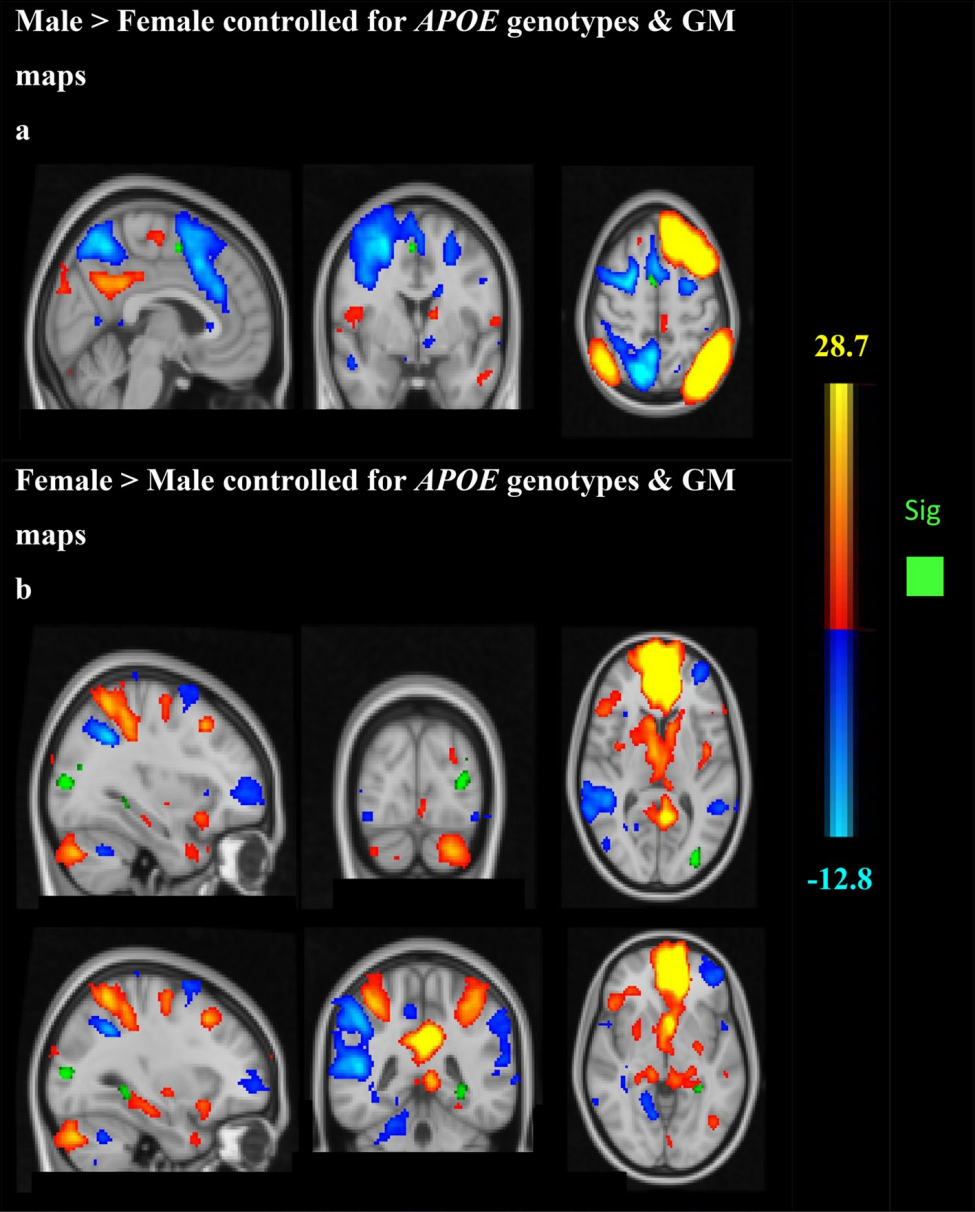
Group-ICA components of the DMN/dorsal–ventral stream and DMN/executive function network. (**a**) Males demonstrating stronger functional connectivity than females between the DMN/dorsal ventral stream (red-yellow/blue) and Juxtapositional lobule cortex (green) (*p* < 0.05 family-wise error [FWE]-corrected). Montreal Neurological Institute (MNI) coordinates x = 4, y = 0, z = 54. (**b**). Females showed stronger functional connectivity between the DMN/executive network (red-yellow/blue) and the lateral occipital cortex (superior & inferior divisions) (x = − 32, y = − 86, z = 8) and the parahippocampal region (x = − 30, y = − 42, z = − 4).

Males showed stronger average functional connectivity between the DMN and the Juxtapositional Lobule cortex, *p* = 0.018, after adjusting for for *APOE* polymorphisms and GM maps. Female subjects showed stronger functional connectivity between the executive network and six different clusters, of which the lateral occipital cortex (inferior & superior divisions) *p* = 0.005 and the parahippocampal gyrus/lingual gyrus, *p* = 0.014 showed the strongest differences, controlled for *APOE* genotypes and GM maps, *p* = 0.03 controlled for *APOE* genotypes, age, cognitive reserve and GM maps (Fig. 2).

Similarly, males showed stronger functional connectivity between the DMN and the Juxtapositional Lobule cortex, *p* = 0.017, after adjusting for *BDNF* genotypes and GM maps. While, females showed stronger functional connectivity between the executive network and seven different clusters, of which the lateral occipital cortex (inferior & superior divisions) *p* = 0.005, and the parahippocampal gyrus/lingual gyrus, *p* = 0.011, showed the strongest differences, controlled for *BDNF* genotypes and GM maps, *p* = 0.03, controlled for *BDNF* genotypes, age, gender, cognitive reserve and GM maps.

### APOE x BDNF interactions

**Figure 3:**
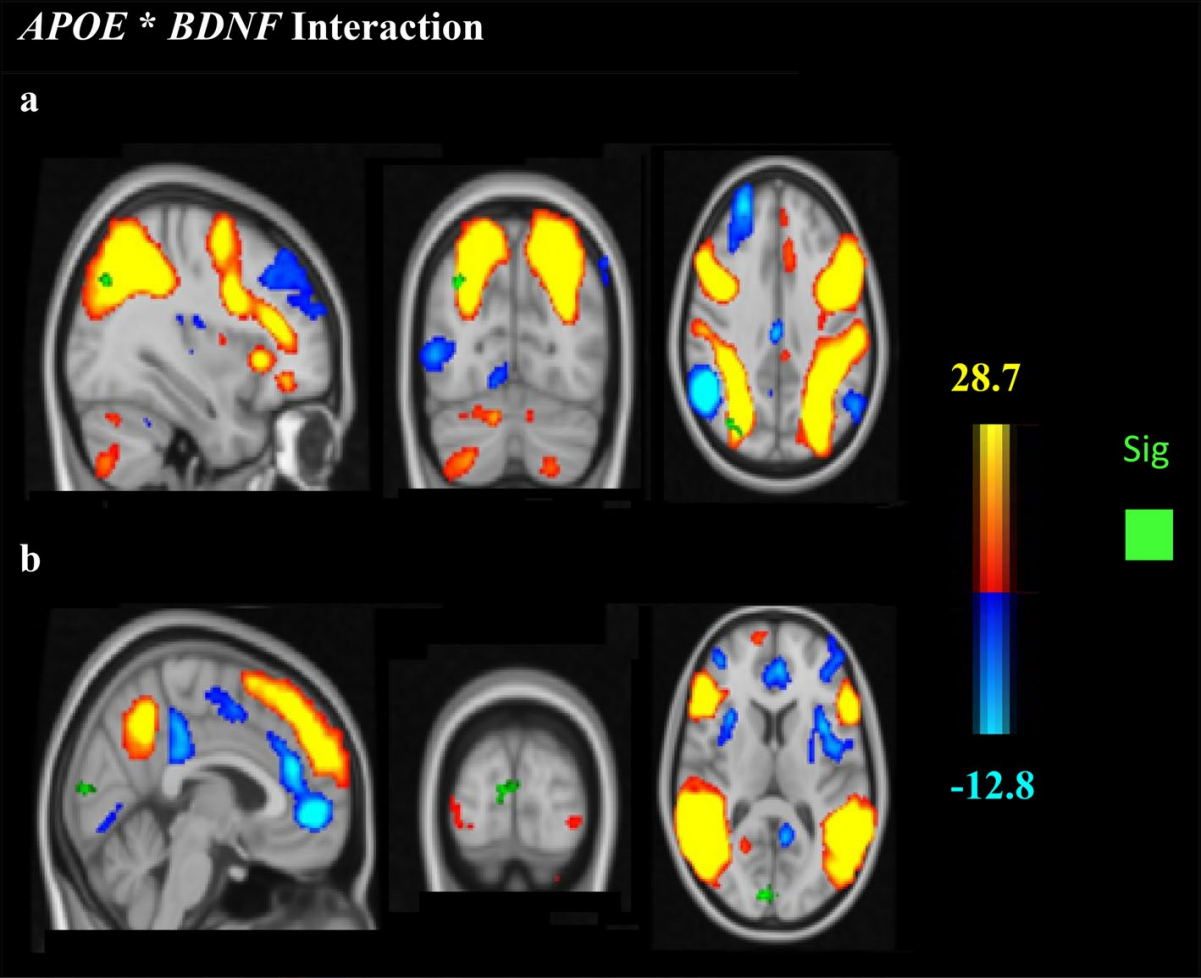
Group-ICA spatial maps of the DAN (**a**) and the dorso-ventral stream/DMN (**b**). (**a**) The findings demonstrate increased connectivity between the DAN and posterior default mode regions (green) in Met/ε3 compared to Met/ε4 (x = 48, y = − 18, z = 2). (**b**) Stronger connectivity was found between the dorsal–ventral stream/DMN and the visual cortex (green) in Met/ε3 carriers than in Val/ε4 carriers (x = 8, y = − 90, z = 8) (*p* < 0.05 family-wise error [FWE]-corrected).

An interaction between the *BDNF* Val66Met and *APOE* polymorphisms was found, with slightly stronger functional connectivity between the DAN and the posterior default mode region in Met/ε3 carriers compared to Met/ε4, *p* = 0.04 (Fig. 3a, Table 3). Stronger functional connectivity was found between the DMN/dorsal–ventral stream and occipital pole in Met/ε3 relative to Val/ε4 carriers, *p* = 0.016 (Fig. 3b, Table 3).

**Table 3:** Apolipoprotein E *(APOE)* * Brain-derived neurotrophic factor (*BDNF)* Interaction.

Cluster Index	Voxels	X (vox)	Y (vox)	Z (vox)	*1-p*	*p*-value	Location
**DAN (Met/ε3 > Met/ε4)**							
1	3	30	25	54	0.953	.047	Lateral Occipital Cortex, superior divison
2	11	27	27	54	0.963	.037	Lateral Occipital Cortex, superior divison
**DMN/dorsal–ventral stream (Met/ε3 > Val/ε4)**							
1	110	41	18	40	0.984	.016	Occipital pole, Intracalcarine Cortex

APOE = Apolipoprotein E; BDNF = brain-derived neurotrophic factor; DMN = Default mode network; DAN = Dorsal Attention Network; GM = grey matter; Met/ε3 = participants not carrying the *APOE* ε4, but at least one copy of the *BDNF* Met alleles; Met/ε4 = participants carrying at least one copy of the *APOE* ε4/*BDNF* Met alleles; Val/ε4 = participants being a Val homozygote and carrying at least one of the *APOE* ε4 alleles; Val**/**ε3 = participants not carrying the *APOE* ε4 allele and being a Val homozygote.

### Cognitive reserve

Cognitive reserve was associated with increased functional connectivity between the DAN and two clusters, left grey matter hippocampal and amygdala regions, *p* = 0.038, and subcallosal cortex, *p* = 0.015 controlled for GM maps, as well as between the DAN and white matter callosal cortex controlled for *APOE*, *p* = 0.03 & *p* = 0.046, controlled for *BDNF*, *p* = 0.016; and between the central executive network and postcentral gyrus, controlled for *APOE* genotypes, *p* = 0.04. Interestingly, functional connectivity decreased with increasing cognitive reserve within the DMN, controlled for *APOE* genotypes, *p* = 0.02, controlled for *BDNF* genotypes*, p* = 0.017 (Table 4, Supplementary Fig. 2).

**Table 4:** Cognitive reserve results of analyses after running dual regressions using GLMs.

Cluster Index	Voxels	X (vox)	Y (vox)	Z (vox)	*1-p*	*p*-value	Location
**Positive correlation between cognitive reserve and functional connectivity, controlled for GM maps**							
DAN							
1	8	59	58	27	0.962	.038	Left GM hippocampus regions, Left GM amygdala regions
2	24	44	76	34	0.985	.015	Subcallosal Cortex
**Negative correlation between cognitive reserve & functional connectivity, controlled for** ***APOE* genotypes & GM maps**							
DMN							
1	2	33	26	46	0.954	.046	Lateral Occipital cortex, superior division
2	6	50	28	44	0.958	.042	Intracalcarine Cortex, Supracalcarine Cortex, Cuneal Cortex, Precuneus
3	80	56	28	44	0.98	.02	Cuneal Cortex, Supracalcarine Cortex, Precuneus,
4	142	42	33	41	0.98	.02	Precuneus, Intracalcarine Cortex
**Positive correlation between cognitive reserve & functional connectivity, controlled for** ***APOE* genotypes & GM maps**							
DAN							
1	3	46	75	38	0.954	.046	WM Callosal Cortex
2	9	44	76	38	0.974	.026	WM Callosal Cortex
**Central Executive Network**							
1	3	12	59	51	0.96	.04	Postcentral gyrus
**Negative correlation between cognitive reserve and functional connectivity, controlled for *BDNF* genotypes & GM maps**							
DMN							
1	2	32	31	43	0.952	.048	Supracalcalinne Cortex, Intracalcarine Cortex, Cuneal Cortex,
2	12	50	28	44	0.963	0.37	Intracalcarine Cortex, Supracalcalinne Cortex, Cuneal Cortex, Precuneus Cortex
3	41	33	26	46	0.971	.029	Lateral Occipital Cortex (superior division)
4	83	56	28	44	0.976	.024	Cuneal Cortex, Supracalcarine Cortex
5	196	35	35	44	0.983	.017	Precuneus, Supracalcarine Cortex
**Positive correlation between cognitive reserve & functional connectivity controlled for** ***BDNF* genotypes & GM maps**							
DAN							
1	21	43	75	35	0.984	.016	Subcallosal Cortex

GLMs = General Linear Models; DMN = Default Mode Network; DAN = Dorsal Attention Network; APOE = Apolipoprotein E; GM = grey matter; BDNF = Brain-derived neurotrophic factor.

## Discussion

In the current study, we investigated cross-sectional differences in resting-state networks between variants of the *BDNF* Val66Met and *APOE* (ε4 & ε3) polymorphisms using resting-state fMRI in an older adult population. In this study, we identified stronger functional connectivity in *APOE* ε3 homozygotes than in *APOE* ε4 carriers, as well as interacting associations between the *APOE* and *BDNF* polymorphisms with respect to connectivity in the DAN and DMN/dorsal–ventral stream. However, there was no significant differences in functional connectivity between *BDNF* Met carriers and *BDNF* Val homozygotes after controlling for GM maps. Last, we found that cognitive reserve was positively associated with functional connectivity within the DAN but was negatively associated with increasing connectivity within the DMN.

Previous research found decreased functional connectivity within the DMN^17,18,32^. We did find significant differences between ε3 homozygotes and ε4 carriers within the DAN after adjusting for GM maps, as well as age, gender, and cognitive reserve. In support Goveas, et al.^17^ also reported disrupted functional connectivity in healthy older ε4 carriers. On the other hand, Dowell, et al.^32^ investigated 37 mid-aged individuals and did not find any *APOE* effects within any resting-state networks, suggesting that alterations within the DMN are not found before the age of 55 years. Furthermore, Dowell, et al.^32^ also reported stronger functional connectivity in the medial visual network, however, only in younger healthy ε3 homozygotes compared to ε4 carriers.

There were no significant differences between *BDNF* Met carriers and *BDNF* Val homozygotes for functional connectivity within the DMN, DAN, and SN. In a previous study, Rodríguez-Rojo, et al.^23^ found that 36 female *BDNF* Met carriers showed poor functional connectivity and reduced memory performance compared to Val homozygotes, using gamma band resting-state functional connectivity. Together, these results suggest that functional connectivity may be influenced by the *APOE* ε4 carriage in healthy older individuals, which possibly is related to AD-pathology, for instance amyloid-beta accumulation in the brain, while the *BDNF* polymorphisms may not have a strong influence on ageing-related functional connectivity alterations in brain networks.

We additionally found interaction effects between the *APOE* and the *BDNF* polymorphisms. Kauppi, et al.^34^ previously described that *APOE* ε3/ε4 alleles and *BDNF* Met/Met alleles were not able to recruit regions of the hippocampus during an encoding task (memory processing). This gene combination was related to a reduction of brain activation in the parahippocampal gyrus and hippocampus, possibly triggering poor memory performance, while non-carriers of both *APOE* ε4 and *BDNF* Met, demonstrated greater activation^34^. The current results from whole brain resting-state functional connectivity analyses revealed slightly stronger functional connectivity within the DAN in Met carriers/ε3 homozygotes relative to Met/ε4 carriers. In addition, we found that Met/ε3 carriers had greater functional connectivity than Val/ε4 carriers between lateral dorsal–ventral stream/DMN and the visual cortex.

Older adults without dementia but with the *APOE* ε4 allele have a relatively high chance of having amyloid-beta deposits in the brain^35^. Hence, the current observations could potentially be related to variations in amyloid-beta burden levels that individuals with the *APOE* ε4 allele in our cohort may carry, however, this is speculative and needs to be further investigated. Chiesa, et al.^36^ observed in longitudinal studies that neither amyloid burden, nor the interaction of *APOE* and amyloid accumulation, altered resting-state functional connectivity within the DMN, proposing that other mechanisms may be involved in resting state alterations in ε4 carriers within the DMN. How the *APOE* status may influence functional connectivity within the *BDNF* Val66Met polymorphisms is also not clear. It has been reported that the expression of the Met allele within ε4 carriers was associated with more amyloid beta load as compared to Val homozygotes, particularly in the precuneus, orbitofrontal cortex, gyrus rectus, and lateral prefrontal cortex^37^. Previous literature has indicated that cognitive performance significantly deteriorates over 3 years in older adult Met/ε4 carriers with higher amyloid-beta load, but not in dementia, in comparison to 10 years for Val/non-ε4 carriers^38^. Conversely, low amyloid-beta levels in healthy elderly individuals was not associated with significant differences in cognitive performance in Met/ε4 carriers, Met/non-ε4 carriers, and Val/ε4 carriers, suggesting that neither the status of *APOE* nor *BDNF* polymorphisms mediated the performance in cognition^38^. Indeed, amyloid beta burden can be detected before any alterations in cognition or behaviour, and commences and accumulates in DMN regions, such as precuneus, medial orbitofrontal, and posterior cingulate areas, disrupting functional connectivity in cognitively healthy individuals^39^ and in AD^7^. Therefore, individuals with amyloid accumulation and mild cognitive impairments (MCI) are more susceptible to develop AD^40^.

Another key aspect of our study was to investigate the relationship between cognitive reserve and functional connectivity. We showed that increased cognitive reserve was positively associated with functional connectivity between the DAN and white matter callosal body, as well as between the DAN and left hippocampus an amygdala. There were further positive correlations between the executive network and postcentral gyrus (primary somatosensory cortex), and between the DAN and the white matter callosal cortex (controlled for *APOE* and *BDNF* genotypes). The subcallosal cingulate brain regions have been found to be involved in respiration control, blood pressure^41^, and in emotional behaviour^42^, and is related to mood disorders such as depression^43^.

Cognitive reserve is a theoretical construct used to describe a mechanism in which the brain compensates or differentially recruits brain networks to maintain cognitive performance despite pathological disturbance^44^. In a more recent review, cognitive reserve is described as being adaptable and flexible, which was related to improved efficiency and capacity of the brain^45^. Cabeza, et al.^46^ on the other hand defined reserve as development of structural processes, in which neural processes enhance cognitive processes more efficiently.

The question why cognitive reserve increased functional connectivity between the DAN and subcallosal cingulate area is difficult to interpret. Previous studies have shown that individuals with more years of education (used as a proxy for cognitive reserve) had enhanced functional connectivity implying that education may promote neural processing, reorganize the brain, and preserve cognitive functions in the process of healthy ageing^47,48^. More recently, Franzmeier, et al.^49^ found that higher cognitive reserve in individuals with amnestic MCI was associated with protection of the functional networks of the anterior DMN-DAN anti-correlation, and also was associated with relatively preserved memory performance. Further, it was observed that people with MCI and high cognitive reserve showed enhanced global functional connectivity within the cognitive control network compared to people with MCI with low cognitive reserve. It was suggested that the brain may compensate in individuals with higher cognitive reserve recruiting other brain regions to accomplish the cognitive functions. Another explanation was that functional connectivity may be enhanced in MCI before pathological alterations of AD manifest and reduce this connectivity^50^. Although, cognitive reserve may influence functional connectivity positively and may have a protective effect on the brain, we also observed a decrease in functional connectivity with increasing cognitive reserve within the DMN and the supracalcarine cortex, intracalcarine cortex, cuneal cortex, and one cluster in the precuneus cortex (controlled for *APOE* and *BDNF*), which was surprising. In contrast, Bosch, et al.^51^ found increased DMN activity in healthy older individuals with higher cognitive reserve. These findings might reflect the different cognitive reserve measurements and different sample sizes. Utevsky, et al.^52^ also reported increased connectivity between the precuneus and the DMN in individuals at rest, however, when individuals were performing a task, enhanced connectivity was found between the precuneus and the fronto-parietal network suggesting high functional flexibility of the precuneus. In patients with MCI or AD, Bozzali, et al.^53^ also described increased DMN connectivity with higher education. Conversely, healthy controls did not show any significant increases in DMN connectivity indicating that education may have moderated functional connectivity in the context of pathological impacts on the brain through compensatory mechanisms and recruiting other brain regions.

A limitation of this study is the small sample size, at 76 participants. A much larger sample size, as reported in Cacciaglia, et al.^54^, may have increased the detection power relative to the *BDNF* and *APOE* polymorphisms. The study also focussed on an island cohort, hence replication in more diverse populations would be required. As noted above, it is also possible that this data may be influenced by the presence of sub-clinical pathological processes in the brains of older adults, such as alterations in amyloid-beta and tau, as well as any potential contributions from cerebrovascular disease. Because 160 volumes (time-points) for resting-state fMRI studies is limited and higher numbers of time-points provide a more accurate estimation, this is a further limitation of this study. Additionally, fieldmaps were acquired but due to technical errors in their acquisition they were unable to be used in the analysis, therefore we used a combination of manual and ICA-based denoising in the preprocessing stage (see Methods). Lastly, the resolution of the fMRI images was slightly lower than comparable studies (3.4 mm).

## Conclusion

To our knowledge, this is the first study to investigate functional connectivity with respect to combinations of *APOE* and *BDNF* variations and relative to cognitive reserve. The carriage of the *APOE* ε4 allele may influence functional connectivity in older adults without dementia. The interaction between the *APOE* and *BDNF* genotypes showing decreased functional connectivity especially in ε4 carriers compared to ε3 homozygotes relative to the BDNF Val66Met polymorphism may be influenced by the *APOE *status. Finally, this study showed that increased cognitive reserve is associated with enhanced or decreased functional connectivity in a network-specific fashion in older adults without dementia.

## Materials and methods

### Study population

#### Participants

For this study, a total sample of 76 healthy participants (53 females, 27 males) aged between 53 and 81 years (Quartile 1 = 58.75 years, median = 63 years, Quartile 3 = 68 years, Interquartile range = 9.25 years) were recruited from the THBP, a prospective cohort study investigating the effect of later-life education on cognitive ageing. A letter was sent out to all THBP participants inviting them to participate in the study. The information about the genetic variants of each consenting participants was known from previous studies and was examined. Volunteers who were invited to participate in this study were specifically selected for a balanced sample of *APOE* ε3/ε3 and ε3/ε4 variants and *BDNF Val66Met* Val/Val and Val/Met variants.

**Figure 4:**
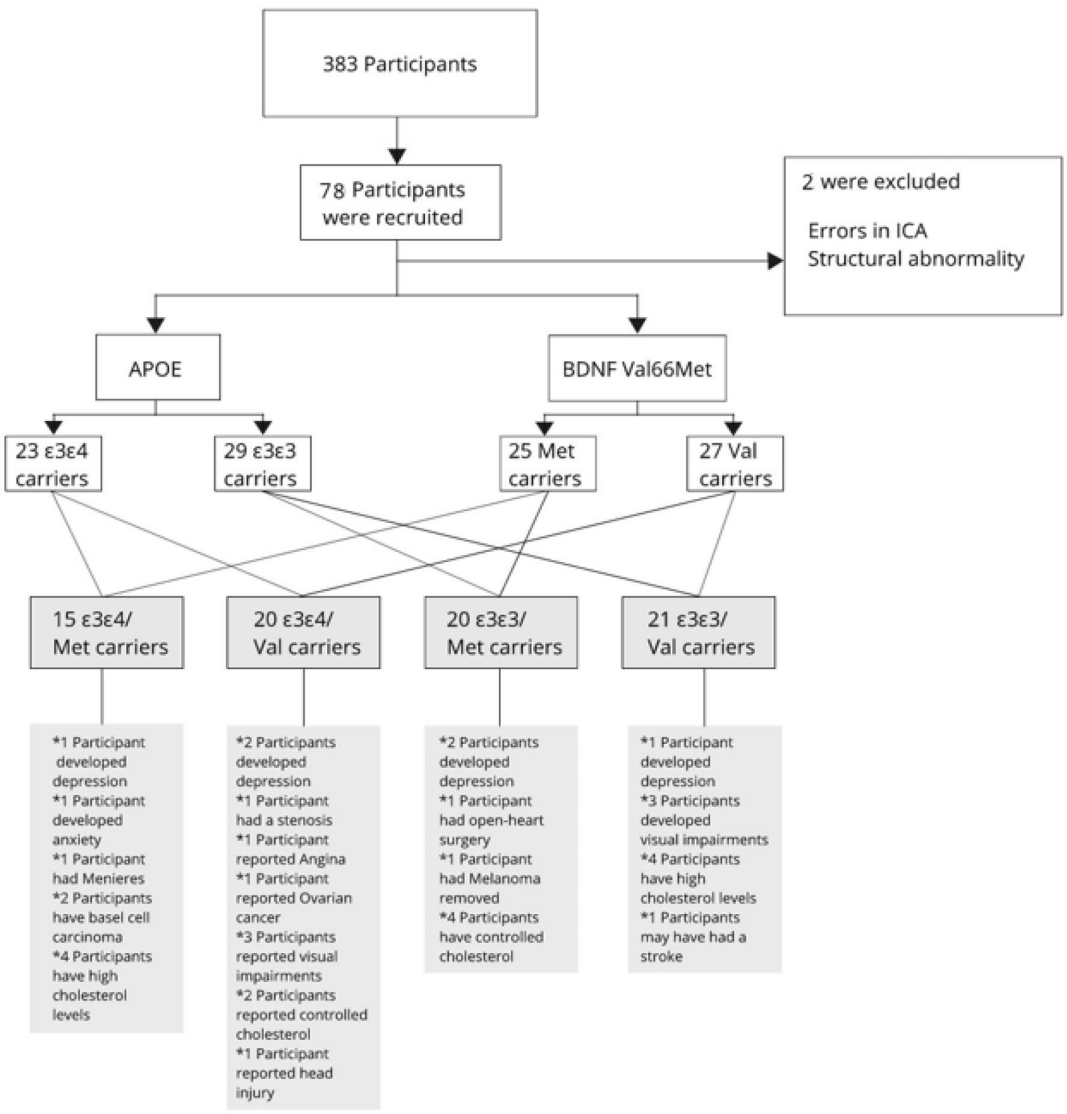
Tasmanian Healthy Brain Project participants, recruitment, inclusion, exclusion, genotypes, and developed medical conditions.

At the time of entry into the THBP (from 2011), the participants were healthy and reported no serious psychological, psychiatric, or medical disorders. Participants with pre-existing conditions were excluded, including cerebrovascular complications, poorly controlled diabetes, poorly controlled hypertension or hypotension, and neurological disorders. A total of 383 adults had commenced in the THBP by December 2012^55^. The current fMRI study participants were recruited between September 2017 and April 2018. Participants have a follow up every two years. The questionnaires examine current background situations such as neurological conditions, psychological conditions, heart-diseases, cancer, colour-blindness, eye vision, blood pressure, cholesterol, head injury, diabetes, kidney- and liver function, and vitamin intake. Within the time of recruitment and scanning (four years in), there was six participants reporting mild to moderate depression, four participants reporting treated cancer, 12 participants reporting higher cholesterol levels of which three are not controlled, six participants reported visual impairments and treatments, and one having a possible stroke. Study protocols for the THBP are described in Summers, et al.^55^. A flow chart representing all participants can be found within Fig. 4.

At the time of recruitment, the participants sample increased from 383 (in 2012) to 460 (in 2015, Ward et al., 2015). For the current study 76 subjects were included in the analysis. Recruitment occurred based on genotypes. Every two years participants are followed up and a medical questionnaire reports all medical conditions the participants experienced.

As part of the larger THBP study, participants chose to be in ‘experimental’ or ‘control’ groups. Participants in the experimental group had previously completed a minimum of 12 months (undergraduate or postgraduate) part-time or full-time university study, while control subjects did not participate in the education intervention. The proportion of experimental and control participants within the ε3 homozygotes (experimental = 33, controls = 9) and ε4 carriers (experimental = 26, controls = 8), and within Val homozygotes (experimental = 31, controls = 10) and Met carriers (experimental = 28, controls = 7) had a fairly balanced proportion and therefore the proportion of experimental and controls were not included as a covariate. Demographic and clinical data for the final sample at baseline are presented in Table 1.

### Procedure

At baseline, THBP participants undertook a comprehensive clinical test battery measuring neuropsychological, cognitive, health, and psychosocial factors^55^, with clinical outcomes overseen by a neuropsychologist. Individuals with significant mental illness and/or impairment of cognitive function were excluded at baseline. This research study has been approved by the Tasmanian Health and Medical Human Research Ethics Committee (Ref No: H0016317) and conducted in accordance with *National Statement on Ethical Conduct in Human Research* (National Health and Medical Research Council of Australia). All subjects provided written informed consent.

#### Cognitive reserve

Cognitive reserve is a hypothetical construct that relates to properties of brain function that are protective in relation to neurodegeneration due to pathological burden. To measure cognitive reserve at baseline, a composite score was derived by factor analysis^58^ from the Wechsler Test of Adult Reading (WTAR; The Psychological Corporation, 2001)^56^, the Lifetime of Experience Questionnaire (LEQ;^57^; and the number of years of prior formal education^55,58^.

The WTAR provides scores about the premorbid intellectual functioning. The task includes 50 words with atypical grapheme to phoneme translation, which the individuals has to pronounce^56,59^. The LEQ provides information about the quality of prior lifetime experiences, such as educational and occupational activity^57^. The number of years in prior formal education were recorded on the Medical Health Status questionnaire^55^. Cognitive reserve was estimated at baseline using a PCA-derived (Principal components analysis) weighted composite score, which were obtained from Ward, et al.^58^.

#### Genotyping

DNA samples from all 78 participants were available for the current study. The collection of DNA samples was carried out with Oragene DNA self-collection kits supplied by Genotek (DNA Genotek Inc., n.d.). *BDNF* Val66Met and *APOE* genotypes were determined through one-step amplified refractory mutation system polymeare chain reaction (ARMS-PCR)^60^ and subsequent gel electrophoresis. For *APOE*, rs429358 and rs7412 were determined by a method reported in Donohoe, et al.^61^. For *BDNF*, Val66Met was determined by the method outlined in Sheikh, et al.^62^. For more information, please review Ward, et al.^25^. Participants were selected for the current study based on their genetic profile: *APOE* ε3ε3 & ε3ε4 and *BDNF* Val/Met & Val/Val.

### Magnetic resonance imaging (MRI)

All brain scans were acquired using a General Electric (GE) Signa 3-Telsa scanner in the Royal Hobart Hospital. Foam pads and headphones were provided to minimize head movement and scanner noise. A Senior Specialist Radiographer supervised the MRI and was responsible for maintaining a safe working environment throughout the MRI session.

Before entering the scanner, participants were instructed to keep their eyes closed, to be relaxed and not to move, and not to fall asleep during data acquisition. The scan took approximately 30 min for each individual.

The imaging sequence was as follows: structural images were acquired using a T1-weighted 3D BRAVO sequence (TR = 1000, TE = 2.53 ms, 256 × 256 × 176 matrix, 1 × 1 × 1 mm voxels). T2-weighted FLAIR (Fluid attenuated inversion recovery) were acquired with 2 mm Iso, TR = 100 ms, TE = 10000 ms, TI = 2250 ms, bandwidth = 83.33 Hz/pixel. Resting state functional MRI scan was acquired using echo planar imaging with a 64 × 64 matrix, 3.438 × 3.438 mm in-plane resolution; slice thickness = 3.4 mm, TR = 2500 ms, TE = 30 ms, Field of view (FOV) = 22 cm.

### Voxel-based morphometry (VBM)

All 76 T1-weighted images were transformed to the correct format. The FSL standard VBM pipeline was followed (https://fsl.fmrib.ox.ac.uk/fsl/fslwiki/FSLVBM/UserGuide) to produce GM maps, which assign a probability of grey matter to each voxel. The processing steps included brain extraction (BET)^70^, segmentation into white matter, grey matter (GM), and cerebrospinal fluid (CSF) volume probability maps using FAST. A study-specific GM template was generated using non-linear registrations^75^ before concatenating them into a 4D image and then smoothed by a range of Gaussian kernels 3.5 mm.

### Functional magnetic resonance imaging (fMRI) Data analysis

#### Resting-state fMRI data pre-processing

Resting-state images were pre-processed using FMRIB Software Library (FSL) v 5.0 (https://fsl.fmrib.ox.ac.uk/fsl/)^31,68^. Each resting-state fMRI dataset consisted of 164 volumes. The first two volumes of each resting-state fMRI image were deleted to avoid potential field inhomogeneities at the beginning of image acquisition. For each dataset MCFLIRT (Motion Correction FMRIB’s Linear Image Registration Tool)^69^ was used to correct for head motion. Brain Extraction Tool (BET)^70^ was used to digitally remove the skull and other non-brain tissue. Additionally, residual non brain tissue was manually removed from T1 structural images using FEAT (FMRIB’s Easy Analysis Tool, ^71^. This ensured appropriate registration from subject- to standard-space (MNI152; BBR;^72^. Each resting-state image was registered to the corresponding structural T1 image (FLIRT)^69,73^ first, and then registered to MNI152 standard space^74^ using non-linear FNIRT tool^75^. Each dataset was resampled to 2 × 2 × 2 mm^3^ resolution in the final MNI152 space. Single datasets of the resting-state functional MRI were temporally high-pass filtered to eliminate slow drifts (cutoff period ~ 100.0 s). In order to reduce noise and preserve spatial information, spatial smoothing with full width at half maximum (FWHM)^76^ of 5 mm Gaussian kernel was used to obtain resting-state networks for each participant.

The pre-processed data was de-noised and analysed using MELODIC (Multivariate Exploratory Linear Optimised Decomposition of Independent Components;^77^ Version v6.00 within FSL to decompose the data into independent components for each subject. Automatic dimensionality estimation was chosen to avoid overfitting. Each individual dataset was manually classified as signal and noise by looking at thresholded spatial maps and temporal power spectra, as well as time courses^78^. No specific software was used to clean the data. Each component labelled as noise was regressed out of the signal using *fsl_regfilt*. All clean data sets were transformed from subject to standard space.

#### Group independent components analysis

To identify resting-state networks, a group-level ICA decomposition was used. Following Feis, et al.^79^ and Damoiseaux, et al.^28^ 25 independent components were extracted using MELODIC. This number of components was chosen to avoid “over-fitting”, which implies that too many components could have caused fragmentation of signal across multiple component maps decreasing the ability to identify the signals of interest. These components reflected the canonical resting-state networks^80^. The group-average maps were used as a template for dual regression (see below). The components of the template were manually classified as resting-state networks or noise artefacts^27,78^. For our analysis, we retained the components of interest: DMN, SN, DAN.

#### Dual regression

The template from the group-ICA was used in a dual regression analysis to estimate differences in connectivity between different genetic groups. An additional dual regression was performed to investigate whether cognitive reserve alone influenced functional connectivity. The dual regression analysis estimates sensitivity to amplitude network activity and to alterations in spatially distributed correlation patterns^81,82^ of resting-state networks. The output of the dual regression produces subject-specific spatial maps and subject-specific data time courses^83^. A FSL tool, called randomise was used for nonparametric permutation inference^84^. With the “–vxl” and “—vxf” options the GM probability maps (GM_mod_merg) were included within the randomise function. General Linear Models (GLM) were used to identify group differences, using the GLM tool in FSL. Statistical significance was evaluated using Monte Carlo permutation-based statistical testing with 5000 permutations at alpha = 0.05^85^. Family-wise error corrections for multiple comparisons using threshold-free cluster enhancement^26^ was used to control Type 1 error rate when investigating differences between *BDNF* Met vs *BDNF* Val, *APOE* ε4 vs *APOE* non-ε4; and the interactions between Met/ε3, Met/ε4, Val/ ε3, Val/ε4. For the genotype’s analyses, cognitive reserve, age, and GM maps were included as covariates to control for individual differences in these parameters. Using the Harvard–Oxford Cortical and Subcortical atlases^86^, probabilistic anatomical labels for local maxima were obtained. All coordinates are reported in MNI space.

### Statistical analysis of demographics

5.4.1. Statistical analyses were performed using Statistical Package for Social Sciences (SPSS) Version 24 from Windows (Chicago, IL, USA). For demographics and clinical characteristics of the different polymorphisms, descriptive statistics were applied. A chi-squared test was performed to compare group differences in gender and education intervention groups across genotypes. A composite score for cognitive function on the domain of language was computed using factor loadings published in earlier work (Ward et al., 2014). The cognitive tests included were Wechsler adult intelligence scale vocabulary and comprehension subtests, and the Boston naming test. To aid interpretability, z-scores were computed by mean-centering and scaling to unit standard deviation (Ward et al., 2014).

## Supplementary Information

Supplementary Information.

## Data Availability

All fMRI data used in this study are archived at the University of Tasmania, Wicking Dementia Research and Education Centre. The author can provide any information on dataset if necessary. Data related to this paper may be requested from the corresponding author.
