# Supplementary material for: Interactive effects of the APOE and BDNF polymorphisms on functional brain connectivity: the Tasmanian Healthy Brain Project
Source: Sci Rep. 2021 Jul 15;11:14514. doi: 10.1038/s41598-021-93610-0 (PMC8282840; doi:10.1038/s41598-021-93610-0)
Supplement: Supplementary file 1 — Supplementary Information. [file 41598_2021_93610_MOESM1_ESM.docx]

**Supplementary Information**

**Interactive effects of the *APOE* and *BDNF* polymorphisms on functional brain connectivity: The Tasmanian Healthy Brain Project**

Manuela Pietzuch^1^*, Aidan Bindoff^1^, Sharna Jamadar^2^, & Prof. James C. Vickers^1^

^1^Wicking Dementia Research and Education Centre, University of Tasmania, Australia

^2^Turner Institute for Brain and Mental Health, Monash University, Australia

*** Corresponding author:**

Manuela Pietzuch

Wicking Dementia Research and Education Centre

University of Tasmania

17 Liverpool Street, Hobart, TAS 7000

Private Bag 417-27

[Manuela.pietzuch@utas.edu.au](mailto:Manuela.pietzuch@utas.edu.au)

Tel: +61 414 515 087

Fax: +61 3 6226 4788

## Supplementary Figures 1-2

| DMN | DMN + part of SN | **48.6**  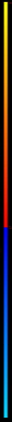  **-13** |
| --- | --- | --- |
| 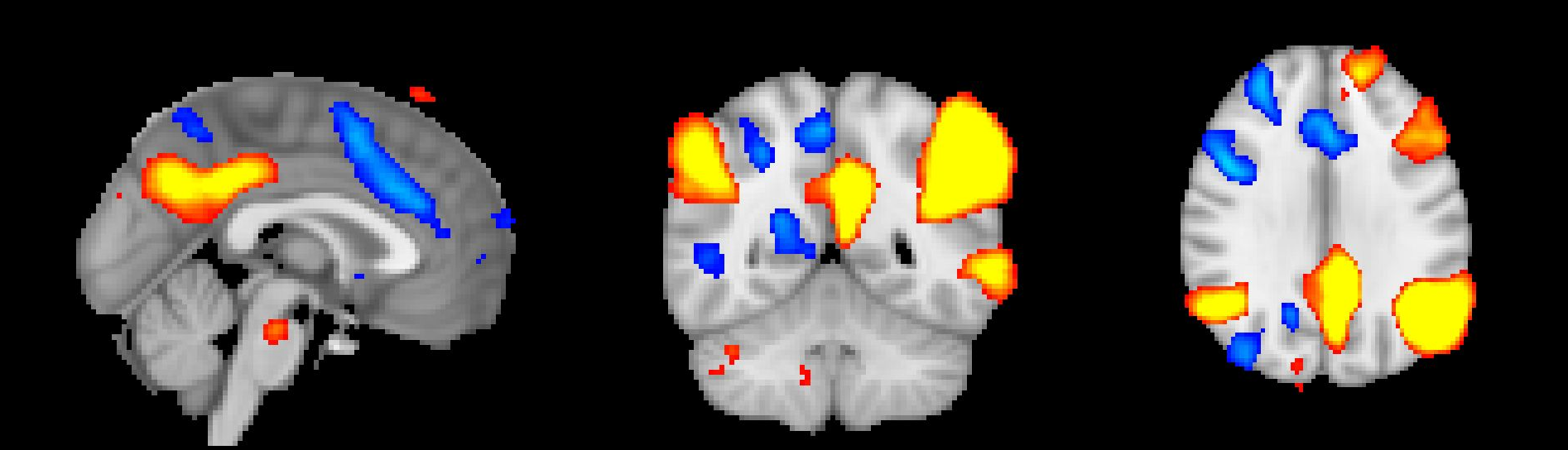 | 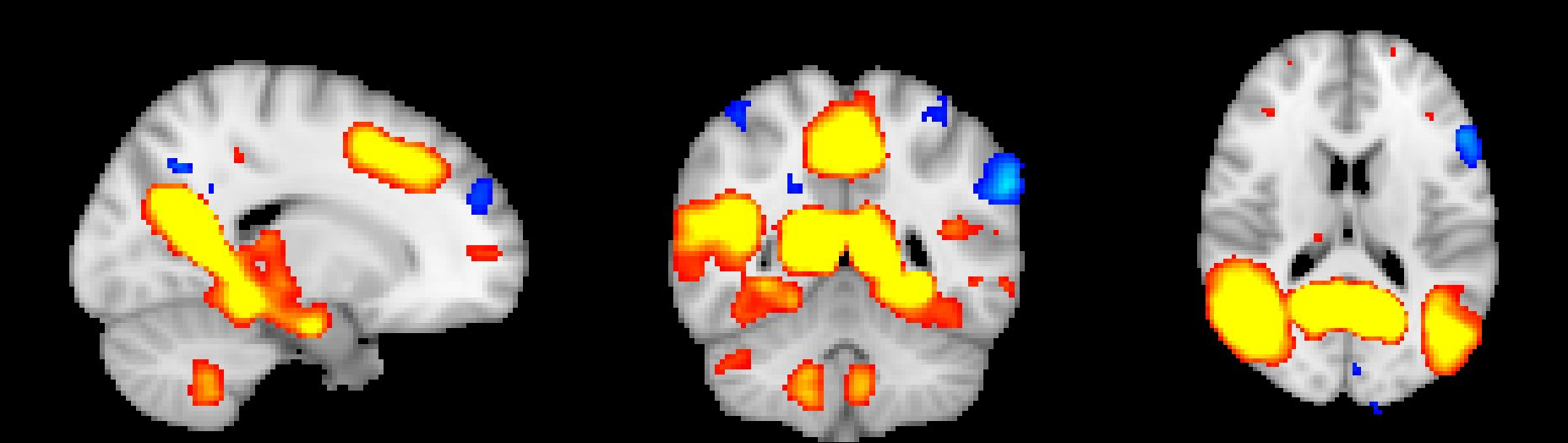 |  |
| Precuneus | Medial visual |  |
| 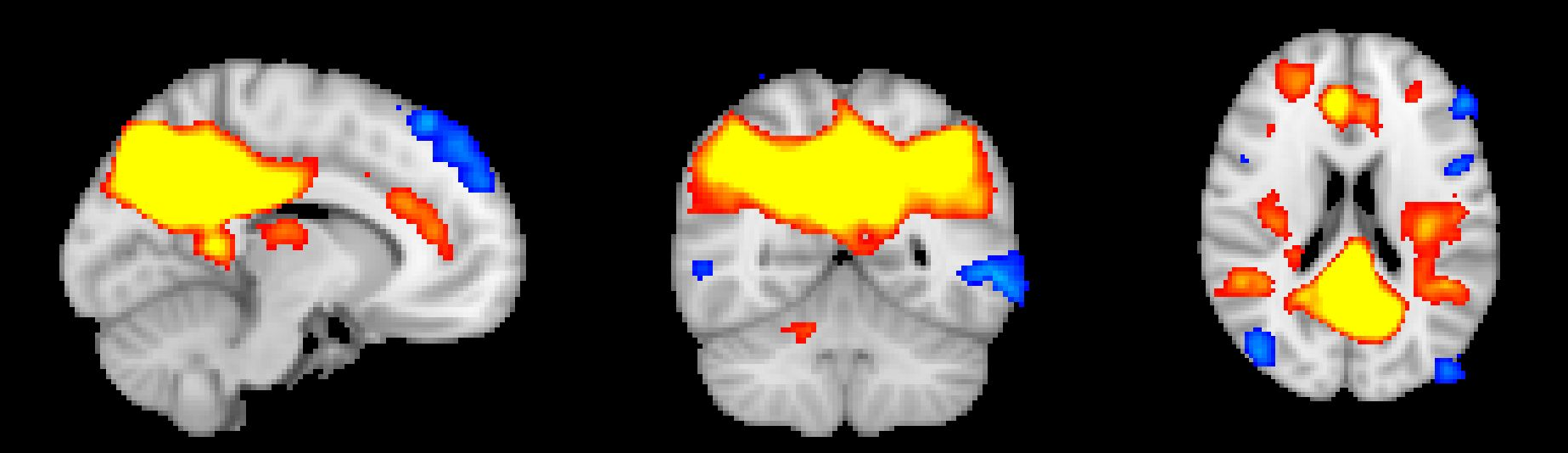 | 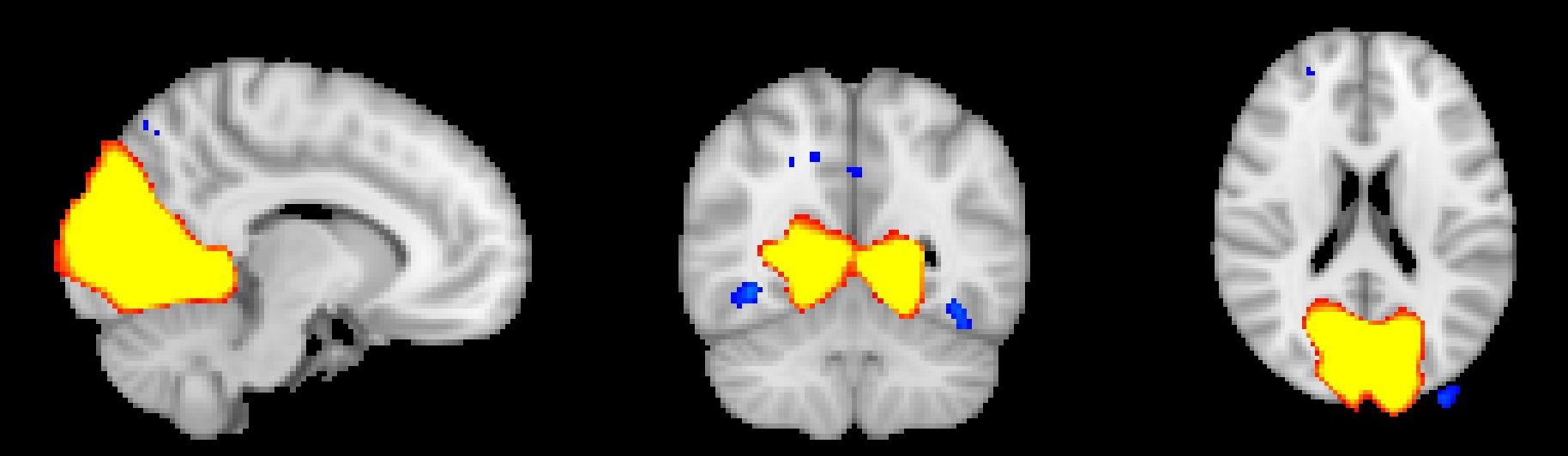 |  |
| L & R Ventral Stream | Lateral visual |  |
| 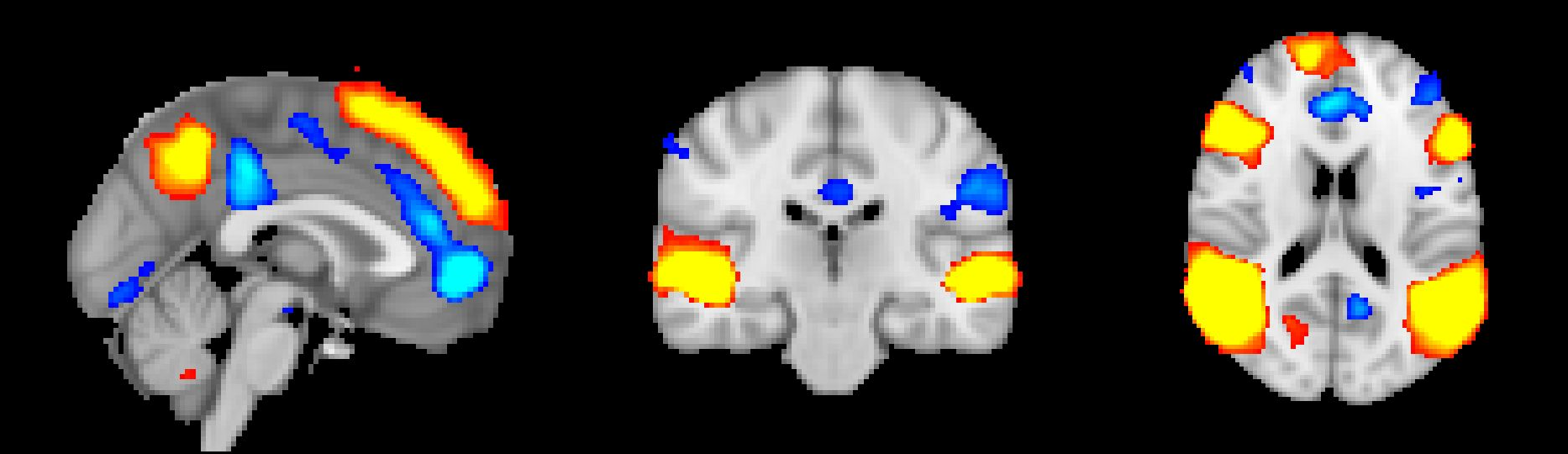 | 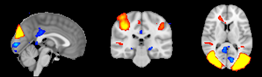 |  |
| R Lateral | Task-positive |  |
| 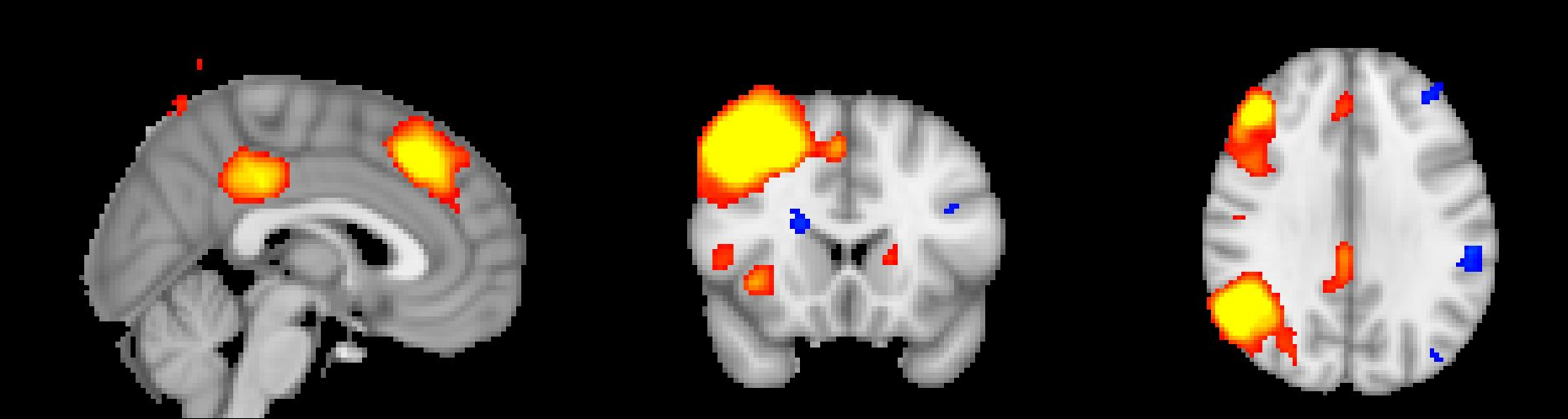 | 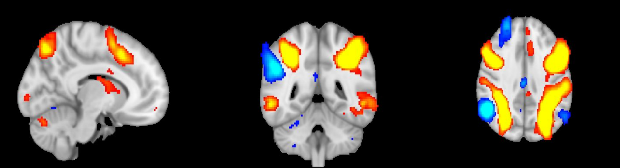 |  |
| Executive Control | Sensory-motor |  |
| 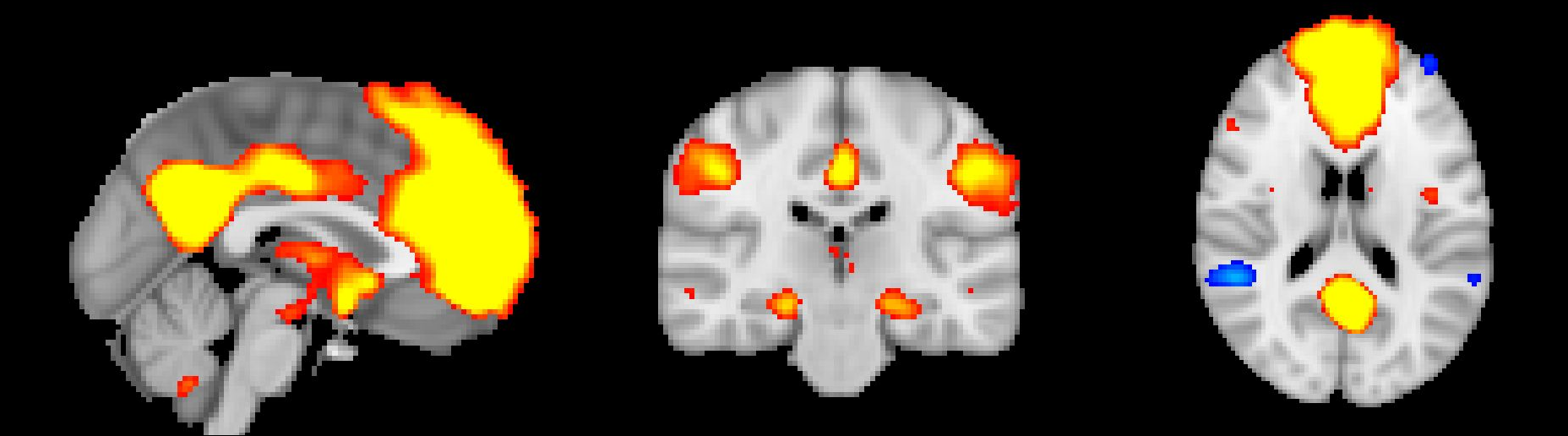 | 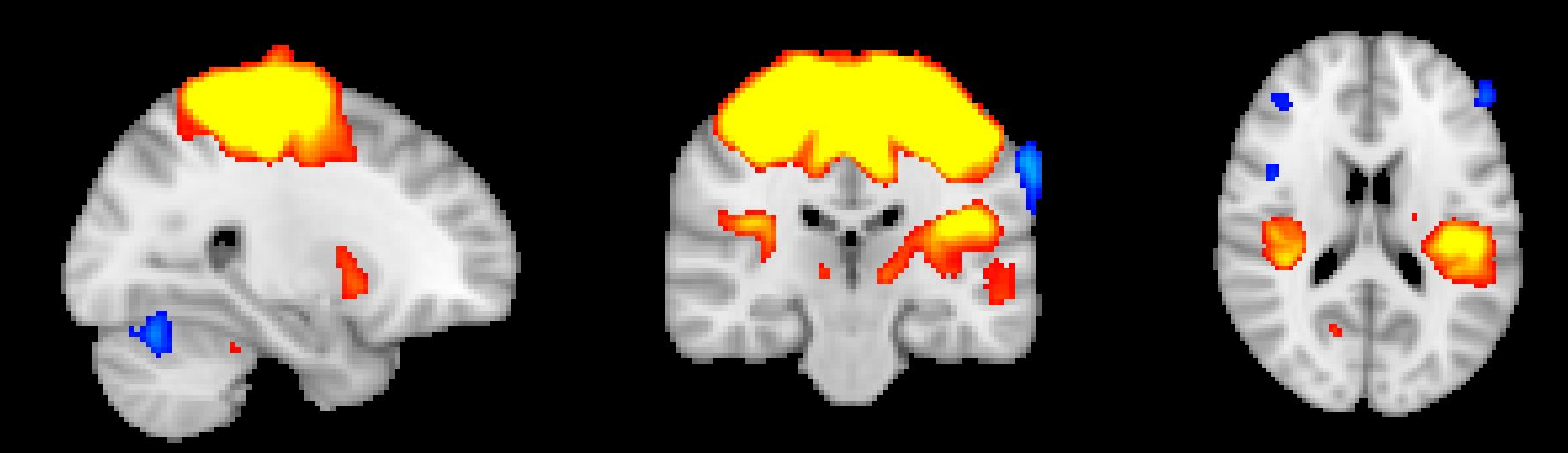 |  |
| Auditory | Cerebellum |  |
| 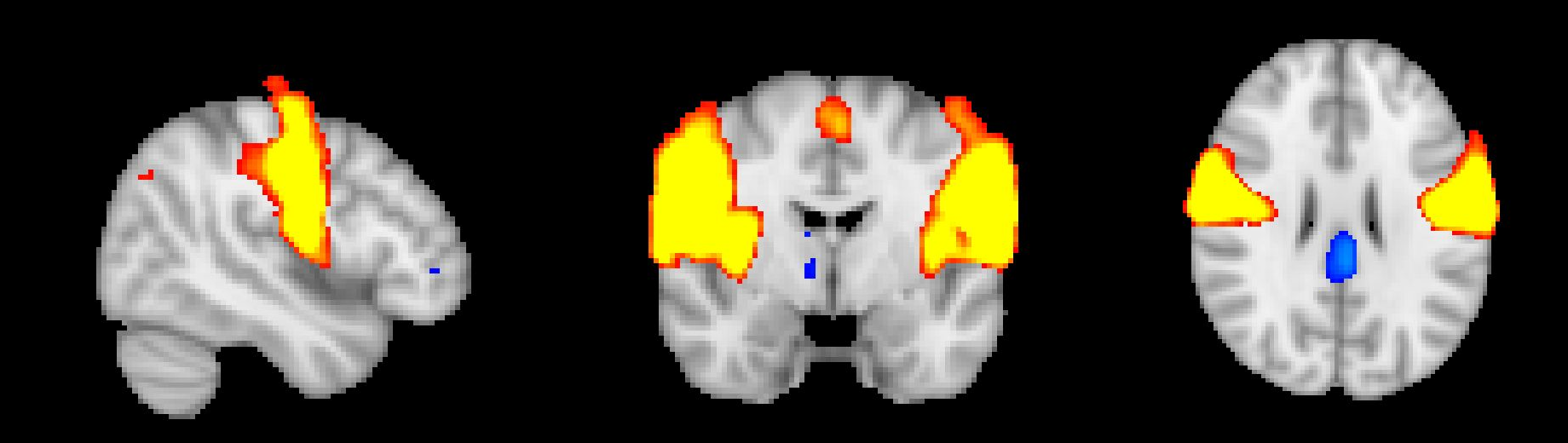 | 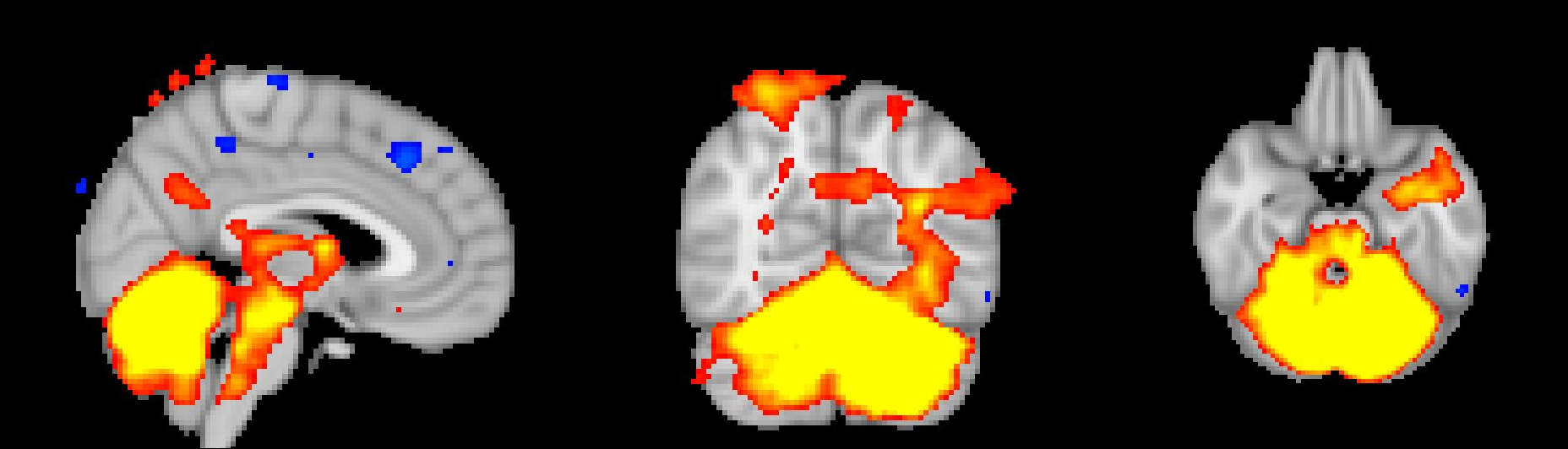 |  |

Supplementary Figure 1. Thirteen resting-state networks from healthy older participants of the Tasmanian Healthy Brain Project. Images depict the percentage of BOLD signal change resulted from the Group-ICA, overlaid on an average high-resolution image transformed into standard space (MNI152). Red to yellow is the percentage signal change showing positive values with this component. Blue are the negative values indicating anti-correlation with this component.

| **Cognitive reserve within the DAN controlled for GM maps only**  ***a***  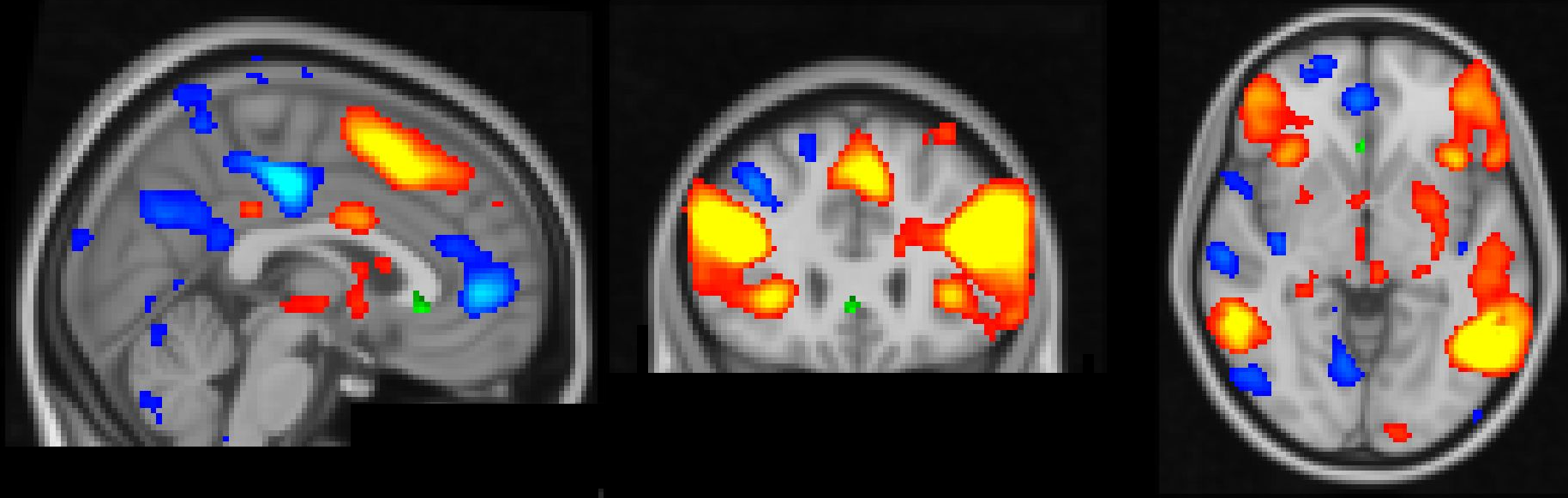  ***b***  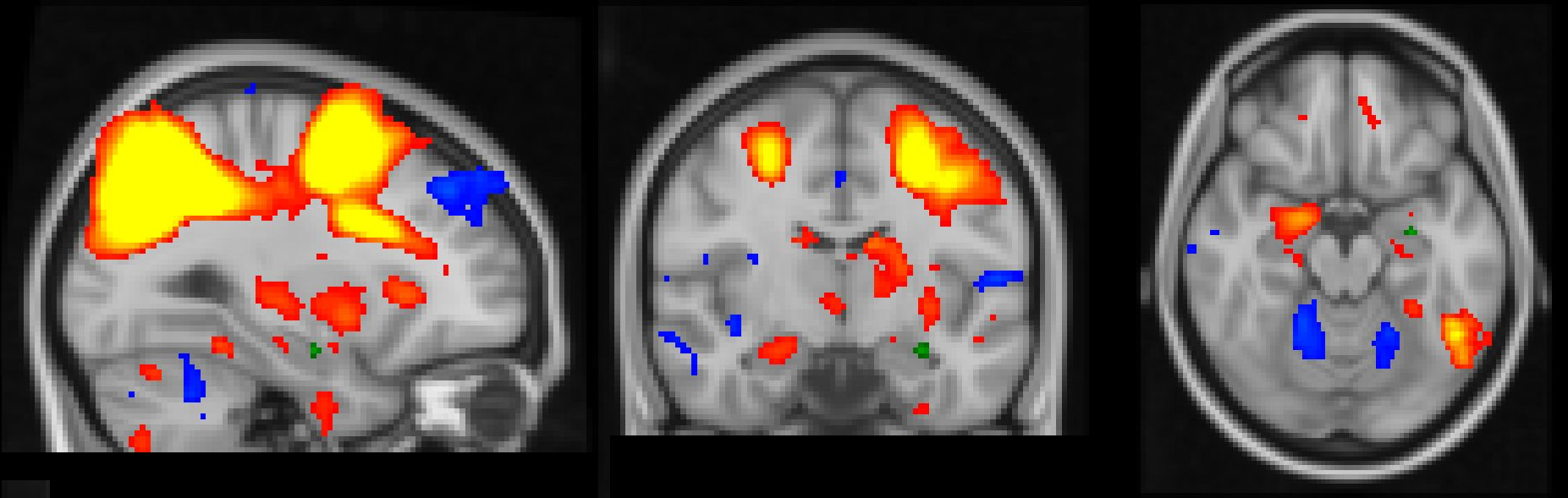 | **33.9**  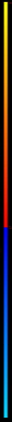  **-9.7** | Sig |
| --- | --- | --- |
| **Cognitive reserve within the DAN controlled for *APOE* & *BDNF &* GM maps**  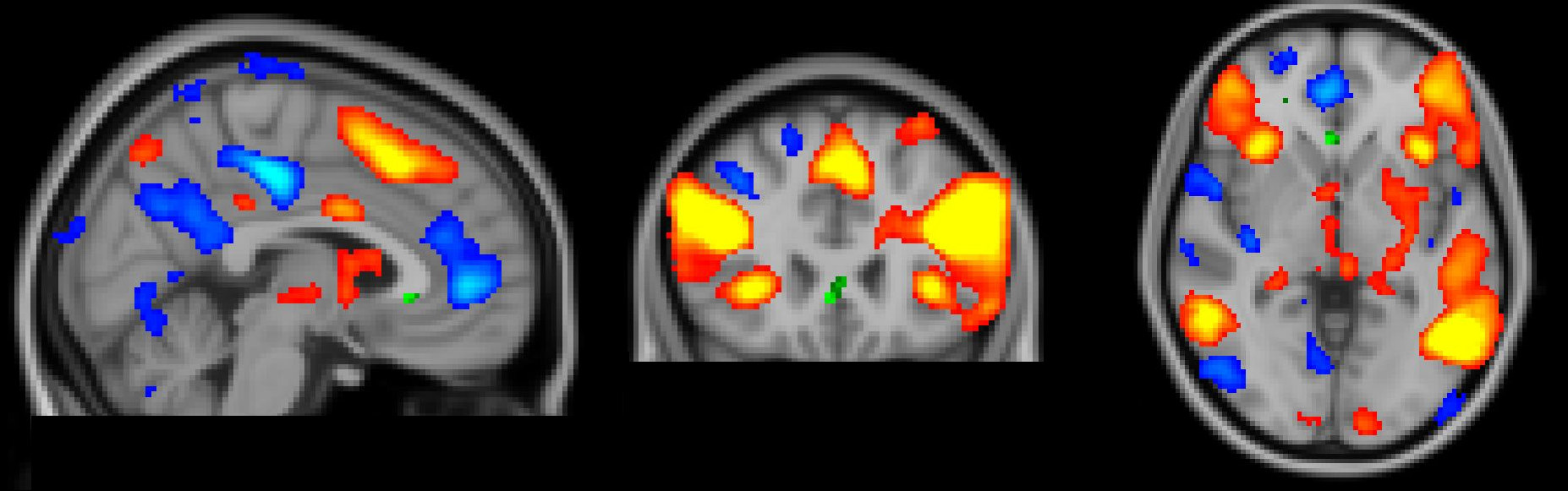 |  |  |
| **Cognitive reserve within the DMN controlled for *APOE* & *BDNF &* GM maps**  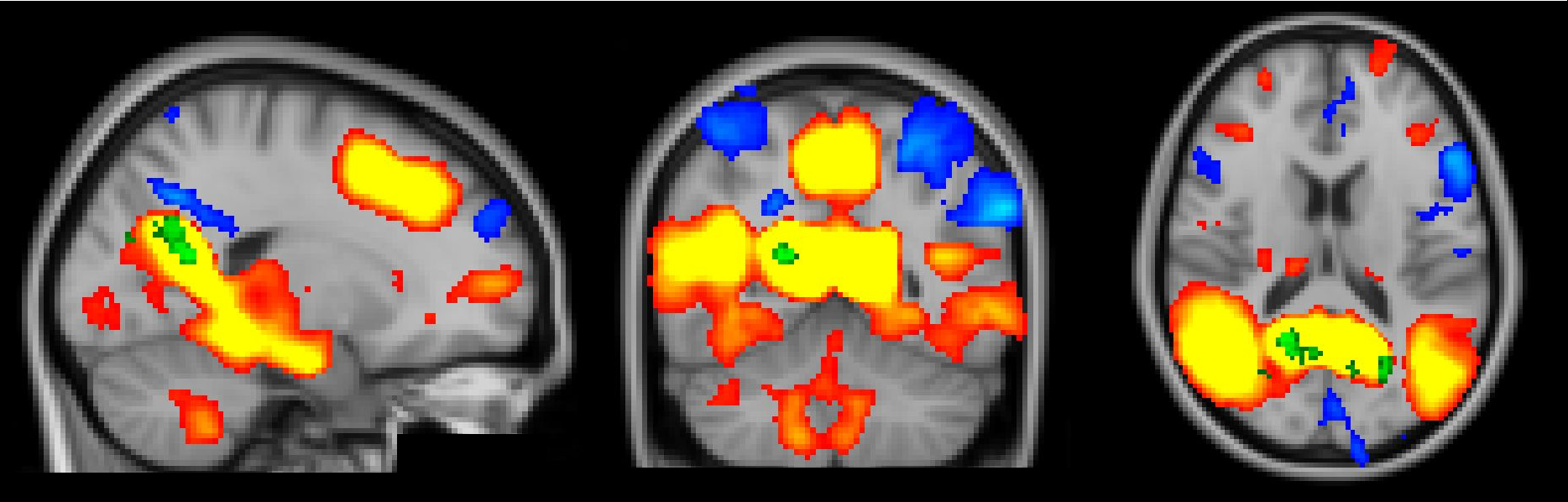 |  |  |

Supplementary Figure 2. Cognitive reserve influences on functional resting networks. Group-level ICA spatial maps of the DAN (top) and DMN (bottom; in red-yellow) overlaid with clusters showing significant influences of cognitive reserve on functional connectivity. Increased functional connectivity associated with increased cognitive reserve in the DAN. MNI coordinates x = -26, y = -8, z = -4 (**a**; left hippocampus/left amygdala). MNI coordinates x = 2, y = 26, z = -4 (**b**; Subcallosal Cortex). Increased functional connectivity associated with increased cognitive reserve within the DAN. MNI coordinates x = -2, y = 26, z = 4 (WM Callosal Cortex). Decreased functional connectivity was associated with increased cognitive reserve in the DMN. MNI coordinates x = 20, y = -56, z = 16 (Precuneus). These results are reported after controlling for *APOE* & *BDNF* genotypes, and GM maps. (All results were *p* < 0.05 family-wise error [FWE]-corrected).
